# Models of Herpes Simplex Virus Latency

**DOI:** 10.3390/v16050747

**Published:** 2024-05-08

**Authors:** Paige N. Canova, Audra J. Charron, David A. Leib

**Affiliations:** 1 Department of Microbiology and Immunology, Geisel School of Medicine at Dartmouth, Lebanon, NH 03756, USA; paige.n.canova.gr@dartmouth.edu; 2 Guarini School of Graduate and Advanced Studies at Dartmouth, Hanover, NH 03755, USA; audra.j.charron@dartmouth.edu

**Keywords:** latency, reactivation, HSV, in vivo and in vitro models, organoids

## Abstract

Our current understanding of HSV latency is based on a variety of clinical observations, and in vivo, ex vivo, and in vitro model systems, each with unique advantages and drawbacks. The criteria for authentically modeling HSV latency include the ability to easily manipulate host genetics and biological pathways, as well as mimicking the immune response and viral pathogenesis in human infections. Although realistically modeling HSV latency is necessary when choosing a model, the cost, time requirement, ethical constraints, and reagent availability are also equally important. Presently, there remains a pressing need for in vivo models that more closely recapitulate human HSV infection. While the current in vivo, ex vivo, and in vitro models used to study HSV latency have limitations, they provide further insights that add to our understanding of latency. In vivo models have shed light on natural infection routes and the interplay between the host immune response and the virus during latency, while in vitro models have been invaluable in elucidating molecular pathways involved in latency. Below, we review the relative advantages and disadvantages of current HSV models and highlight insights gained through each.

## 1. Introduction

### 1.1. Prevalence and Pathogenesis

Herpes simplex virus (HSV) includes two neurotropic subtypes (HSV-1 and HSV-2) that enter the body through mucosal surfaces and establish lifelong infections in neurons of the peripheral and central nervous systems (PNS and CNS). A majority of HSV infections are acquired during adolescence as interpersonal intimacy begins, and viral prevalence increases with age. By age 50, HSV-1 affects nearly 70% of the global population, while HSV-2 affects approximately 13% of the population [1,2,3]. HSV-1 is slightly more prevalent in females, while HSV-2 seroprevalence is doubled in women compared to men. This is thought to be a result of the increased efficiency of sexual transmission from men to women [1,2]. Although HSV-1 is typically associated with oral infections and HSV-2 is associated with genital infections, both subtypes have the ability to cause oral or genital lesions [4,5]. In fact, current trends in developed countries indicate that genital infections are now more commonly caused by HSV-1 than HSV-2 [6]. 

While HSV is most commonly associated with herpetic skin lesions, it also causes more serious disease, including corneal keratitis and neovascularization, encephalitis, and meningitis [7,8,9,10,11]. Ocular presentation, a complex pathogenic interplay between lytic infection and adaptive immune activation, makes HSV the leading cause of infectious blindness in the United States [12]. In addition, although rare (with an incidence of 1 in 10,000 live births), neonatal HSV-1 infections are devastating, particularly in babies born to mothers who did not develop protective humoral immunity prior to parturition [1,13,14]. A recent cohort of publications have drawn attention to a correlation between HSV infection and neurodegeneration in diseases such as Alzheimer’s disease, multiple sclerosis, and Parkinson’s disease. HSV-1 genomes are found with increased frequency in the brains of multiple sclerosis patients and are co-localized with amyloid-beta (Aβ) deposits in Alzheimer’s disease patients [15,16]. Moreover, HSV-1 infection results in the accumulation and abnormal distribution of Aβ in neurons, promoting disease progression [17,18]. As HSV intercepts cellular pathways that are critical to neuronal health (apoptosis, autophagy, mitochondrial function, DNA damage, and oxidative stress response), these modifications are likely to aid in HSV persistence leading to neuronal damage [19].

### 1.2. HSV Classification and Structure

HSV-1 and HSV-2 are human viruses of the family Herpesviridae, subfamily Alphaherpesvirinae, which also includes the varicella zoster virus (VZV), the causative agent of chicken pox and shingles, and the swine pathogen pseudorabies virus (PRV) [20,21]. The HSV genome consists of a linear double-stranded DNA molecule of ~150 kb contained within a capsid [22]. While most of the ~80 HSV genes are haploid, others are encoded within inverted repeats and are thus diploid [22,23]. Surrounding the capsid, tegument proteins are packaged for co-delivery with the genome. The genome, capsid, and tegument are enclosed within the viral envelope, which is studded with numerous glycoproteins specialized for host cell binding, membrane fusion, and immune evasion [24].

### 1.3. HSV-1 Lifecycle: Lytic Infection

The primary infection of HSV-1 typically occurs at mucosae such as the eyes, mouth, and genitals. Once the mucosal surface is breached, viral replication and epithelial cell lysis ensure its spread to the surrounding epithelium and underlying peripheral neurons. Virions that bind to the neuronal cell surface glycoprotein nectin-1 enter the axons through direct membrane fusion or receptor-mediated endocytosis [25]. Genome-filled capsids are then transported retrogradely by kinesin motors to the neuronal soma, where the linear viral genome is injected forcibly into the nucleus [26,27]. Once there, it circularizes and becomes associated with modified histones [28,29]. At this point, the genome may be silenced, establishing latency, or may be transcribed and replicated for lytic viral production. During lytic infection, viral gene expression is initiated by a complex consisting of the tegument protein VP16 and the host transcriptional co-factors HCF-1 and Oct-1, amongst others [30]. Viral gene transcription and DNA replication occur through the sequential activation of gene classes, starting with the immediate-early (IE) genes (ICP0, ICP4, ICP22, ICP27, and ICP47), which, in turn, activate the early (E) genes and, finally, the late (L) genes. In addition to transcriptional activation, the IE gene products also inhibit cellular anti-viral defenses [31,32,33,34,35,36,37,38,39,40,41,42]. The E gene products are primarily involved in viral genome replication, while L gene products are important for the virion structure, egress, and immune evasion [43]. While the lytic cycle occurs in both epithelial cells and neurons, latency is unique to neuronal infection [44]. Viral reactivation from latency is initiated by pleiotropic stresses that tip the balance of viral-versus-host control. Once the lytic cycle is re-entered, newly assembled virions travel anterogradely within the axon to reach the initial or new site of epithelial mucosa within the dermatome, where another round of replication and spread occurs.

### 1.4. HSV-1 Lifecycle: Latent Infection

**Figure 1:**
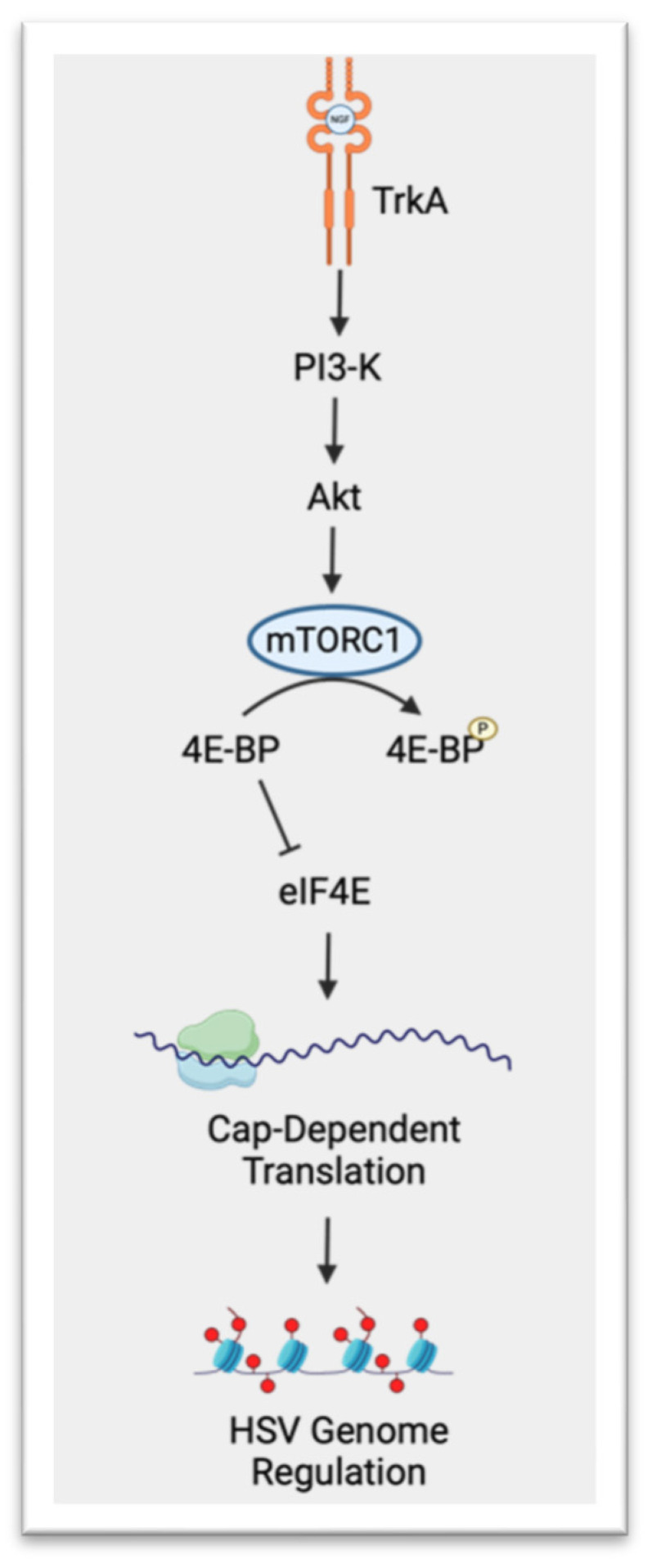
HSV lytic gene regulation by mTORC1. Unphosphorylated eIF4E-binding protein (4E-BP) associates with eIF4E and inhibits recruitment of the 40S ribosome to mRNA, which suppresses translation. Upon binding of nerve growth factor (NGF) to the TrkA receptor tyrosine kinase, phosphatidylinositol 3-kinase (PI3-K) and then Akt are activated. Akt activates the mTORC1 complex, which inactivates 4E-BP through phosphorylation, allowing for translation and protein synthesis to occur. Newly synthesized proteins possibly aid in maintaining HSV latency through epigenetic chromatin modification. Created with BioRender.com.

The sporadic reactivation of HSV from latency is a key factor in the lifelong duration of HSV infections. There are neither effective vaccines against HSV nor anti-viral agents that can completely prevent the establishment, maintenance, or reactivation of latency. Owing to the non-renewing and essential nature of neurons, immune targeting of the infected neuron appears to be specialized and cell-sparing [45]. The regulation of latency has been linked to the neuronal phosphatidylinositol 3-kinase (PI3-K)/Akt signaling pathway [46], illustrated in Figure 1. This pathway is vital for neuronal functions, including synaptic plasticity, axonal growth and regeneration, and long-term potentiation [47,48,49]. Nerve growth factor (NGF) binding to the TrkA receptor tyrosine kinase initiates the activation of PI3-K and Akt, which then activate mTOR, consisting of the mTORC1 and mTORC2 complexes. The mTORC1 complex is considered a regulator of neuronal homeostasis due to its ability to both detect the availability of growth factors and nutrients, and direct responses that regulate translation, autophagy, cell growth, and metabolism [50,51]. Each of these processes requires protein synthesis, which is regulated through the mTORC1 substrate, eIF4E-binding protein (4E-BP) [51,52]. 4E-BP is a translational repressor that inhibits 40S ribosome recruitment to mRNA by associating with the cap-binding initiation factor eIF4E [53]. Its inactivation through phosphorylation by mTORC1 allows for the continuous translation and synthesis of proteins necessary to suppress HSV reactivation and maintain latency [50,52,53]. This regulation of protein synthesis by mTORC1 and 4E-BP may impact epigenetic chromatin modifications, expression of viral-encoded microRNAs, localization of HCF-1, and repression of stress-response pathways as mechanisms to promote HSV latency [54,55,56].

**Figure 2:**
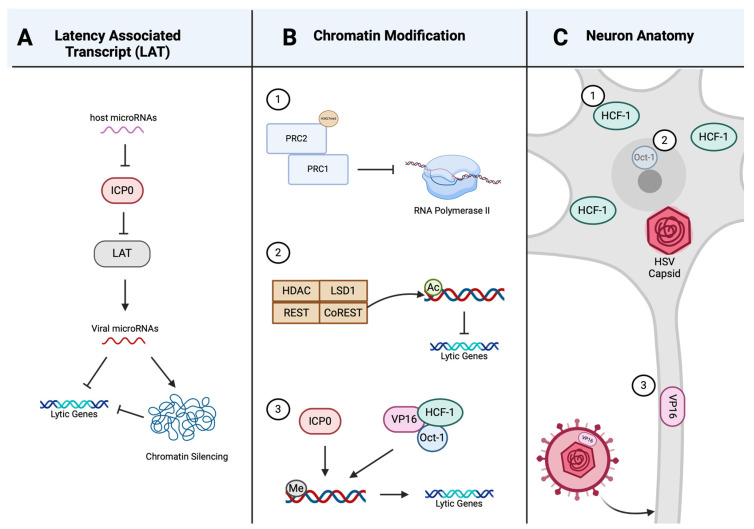
Mechanisms involved in regulating HSV latency. (**A**) Host microRNAs can restrict lytic gene expression by repressing ICP0, which then is unable to suppress latency-associated transcripts (LATs). Viral microRNAs transcribed from LAT can then restrict lytic gene expression directly or through chromatin silencing. (**B**) Chromatin modification can regulate lytic gene expression in three ways: (1) suppressing RNA polymerase II-mediated transcription through the *Polycomb* group (PcG) repression complexes PRC2 and PRC1, (2) lytic gene-dependent deacetylation and demethylation of the HDAC/LSD1/REST/CoREST (HLRC) complex, and (3) promoting lytic gene expression through the removal of histone methylation by ICP0 or the VP16/Oct-1/HCF-1 complex. (**C**) Distinguishing characteristics of neurons can influence lytic gene expression through the limited formation of the VP16/HCF-1/Oct-1 complex in the nucleus of neurons in three main ways: (1) the host transcription factor HCF-1 is localized exclusively in the cytoplasm in sensory neurons, (2) decreased expression of the host transcription factor Oct-1 in neurons, and (3) decreased VP16 reaching the neuronal nucleus due to the dissociation of VP16 from the viral capsid during long-distance transport through the axons. Created with BioRender.com.

While viral lytic gene expression is silenced, the non-protein coding RNAs termed latency-associated transcripts (LATs) accumulate during latency. The primary 8.3 kb LAT is spliced into a stable 2 kb intron and further spliced into a 1.3–1.5 kb intron [57]. This region of LAT is complimentary to the lytic IE gene encoding infected cell protein 0 (ICP0), a gene non-essential for viral replication, but pivotal for reactivation [57,58,59,60]. LAT may regulate latency via antisense RNA binding and the destruction of ICP0 transcripts [57]. Additionally, cellular and viral microRNAs suppress the expression of IE genes involved in the onset of lytic replication [61]. Viral microRNAs are transcribed by the LAT promoter and may target either viral lytic genes or host genes involved in transcriptional regulation and chromatin silencing, as depicted in Figure 2A [61,62]. The viral microRNA miR-H2-3p suppresses ICP0 expression though antisense pairing to the coding region of ICP0 (akin to LAT) [63]. Host microRNAs also regulate lytic replication and latency in a similar manner, restricting viral IE gene expression (Figure 2A) [64]. The neuron-specific microRNA miR-138-5p represses the expression of ICP0 of both HSV-1 and HSV-2 [65,66]. miR-138-5p also restricts the expression of host transcription factors Oct-1 and FOXC1, both of which promote viral replication [67]. Oct-1 is part of a complex essential for initial IE gene expression, and FOXC1 modulates the heterochromatin structure of viral genes to promote lytic gene expression [67].

Viral gene expression is regulated, in part, through histone modification of lytic gene promoter chromatin. Viral lytic genes are silenced when their promotors are enriched in repressive chromatin histones such as H3 di- and tri-methylated lysine 9 (H3K9me2/3), lysine-27 (H3K27me3), and reduced acetylated histones [68]. While H3K9me3 is associated with stable heterochromatin through its interaction with heterochromatin protein 1 (HP1) bound to nucleosomes, H3K27me3 is a marker of epigenetic silencing by the *Polycomb* group (PcG) repression complexes PRC1 and PRC2 [69,70]. The PcG-mediated chromatin repression occurs through PRC2, which contains the H3K27me3 methyltransferases subunit, recruiting PRC1 to suppress RNA polymerase II-mediated transcript elongation (Figure 2B) [71]. Another complex consisting of HDAC/LSD1/REST/CoREST (HLRC), which silences neuronal genes in non-neuronal cells, can also repress viral E and L lytic genes through deacetylase and demethylase activities [72,73,74,75]. ICP0 is able to counter HLCR gene repression through displacing HDAC1; thus, silencing of viral genes through the HLRC complex can be regulated by ICP0 expression (Figure 2B) [71,73,76]. While the REST/CoREST repressor complex is thought to limit viral replication and affect latency establishment, it does not play a role in reactivation [73]. The repression of viral lytic genes can be reversed by replacing the histone modifications H3K9me3 and H3K27me3 with trimethyl histone H3 lysine-4 (H3K4me3) chromatin activating marks [68]. This reversal of chromatin markers can be initiated by either ICP0 or the VP16/Oct-1/HCF-1 complex (Figure 2B) [77,78].

Some authors have proposed that it is the unique anatomy and physiology of neurons that drives cell-selective latency [64,65]. VP16 dissociates from the viral capsid during entry and transport along the neuronal axon, thus limiting the amount of VP16 that reaches the neuronal cell body (Figure 2C) [79]. Not only does less VP16 reach the nucleus of neurons (relative to non-neuronal cells), but in sensory neurons, HCF-1 localizes exclusively to the cytoplasm and Oct-1 expression is low (Figure 2C) [80,81]. This results in less VP16/HCF-1/Oct-1 complex within the neuronal nucleus and decreased lytic gene expression, promoting latency. Furthermore, neuronal specific factors differentially regulate the IE promoters of ICP0 and ICP27 [82,83]. While neurons have limited intrinsic immunity to HSV-1, they do respond to extrinsic cues, thus affecting latency [39]. Interferons promote latency establishment in dissociated ganglia cultures and impact reactivation, while neuronal tissue-resident CD8^+^ T-cells may suppress reactivation from latency through directed targeting of infected neurons with granzyme B and IFN-γ [84,85,86]. Our understanding of the mechanisms that regulate latency establishment, maintenance, and reactivation was obtained through a variety of in vivo, ex vivo, and in vitro models [56,87,88]. The limitations and advantages of the most common models used to study HSV latency are discussed below.

## 2. In Vivo Models of Latency

A variety of animal models have been developed to mimic different anatomical manifestations and stages of HSV infections, as well as for the study of different stages of the HSV lifecycle (reviewed in [89]). In this section, summarized in Table 1, we discuss and compare many of these models.

### 2.1. Mice

The mouse is a commonly used animal model of HSV infection. This is largely due to well-defined inbred and transgenic strains; cost efficiency due to small size, minimizing reagent expenditure and per diems; and the wide availability of immunological probes and reagents [92,93]. Although these are key advantages, HSV infection in mice has some drawbacks, at least in part due to several important differences in immune responses between mice and humans. First, viral peptides are displayed at the infected cell surface by major histocompatibility complex class I (MHC-I) molecules. In humans, the HSV-1 ICP47 protein inhibits the transporter associated with antigen processing (TAP), preventing peptide loading and presentation by MHC-I and, thus, T-cell detection of infected cells [94]. ICP47, however, functions 100-fold less efficiently in mice, resulting in increased antigen presentation and robust immune detection [95]. Second, the affinity of host immunoglobulin G (IgG) for gE, the HSV IgG Fc receptor protein, is higher in humans than in mice [96]. Therefore, human, but not mouse, IgG is bound with high affinity to gE on extracellular virions and gE-coated HSV-infected cells, considerably increasing antibody efficacy in mice [96]. Third, the murine version of the transcription factor Oct-1 has a relatively weaker interaction with VP16 than the human form, diminishing initiation of lytic viral gene expression [97]. Fourth, and possibly as a consequence of these differences, HSV-1 spontaneously reactivates from latency in humans, but not commonly in mouse infections [98]. Fifth, in humans, the HSV entry coreceptor nectin-1 is expressed on the surface of vaginal epithelial cells throughout the menstrual cycle [99]. In mice, however, nectin-1 is expressed only during the diestrous and proestrous phases. Hormone manipulation is therefore necessary to ensure genital HSV infection in the mouse [100]. The progesterone treatment itself induces thinning of the vaginal epithelium, causing a 100-fold greater susceptibility to genital HSV-2 infection in the diestrous phase and possibly reduced immune protection to HSV-2 [101]. Finally, the lack of spontaneous reactivation means that it is not possible to study horizontal (peer-to-peer) or vertical (parent-to-progeny) HSV transmission in mice [100]. These difference in host–virus interactions mean that mice are not always reliable as predictive models for vaccine development [102]. While preclinical HSV vaccine development has been largely based on the mouse model, none yet has succeeded [93]. This emphasizes the need for other animal models for the study of HSV latency and reactivation, and immunologic or pharmaceutical protection from HSV.

### 2.2. Rabbits

Like humans, rabbits experience spontaneous reactivation of latent HSV [112]. Typically used for ocular infections, rabbits shed HSV-1 through tears and have recurrent ocular lesions resembling human infections [110,111]. As a model of HSV-1 infection, rabbits have the advantage of ocular morphology and mucosal immunity akin to humans, as well as similar T-cell-mediated ocular disease presentation [93,113,114]. Their larger size makes rabbits more amenable to in vivo, in situ, and ex vivo studies than mice [93]. Immunological reagents are not as plentiful as those for mice but are readily available. While rabbits’ ocular mucosal immune system is known to be similar to that of humans, the rabbit systemic immune system is not yet well characterized. To address this issue, a human leukocyte antigen (HLA) transgenic rabbit expressing human HLA class I molecules was developed to better mimic HSV infection in allowing an immune response to human HSV-1 CD8^+^ T-cell epitopes [115]. While not broadly used, this model, coupled with spontaneous reactivation, makes rabbits a suitable model to study vaccine efficacy, perhaps following initial screening in mice.

### 2.3. Guinea Pigs

Since the 1980s, the guinea pig has been the most commonly used clinically relevant model to study genital herpes infections caused by HSV-2. At low doses of HSV-2, guinea pigs present similarly to a human infection with viral shedding, spontaneous reactivation, and recurrent lesions [107,108]. Like humans, guinea pigs with intravaginal HSV-2 infections develop self-limited primary vulvovaginitis and latency in the sacral ganglia and lumbosacral dorsal root ganglia [109]. Unfortunately, due to limited immunologic reagents and inbred strains, the scope of immune effector mechanisms is currently not well characterized [93]. While guinea pigs have similar immune system characteristics to humans, they have incompatibility between endogenous T-cells and HLA-restricted T-cell epitopes. They therefore cannot be used to model protective efficacy of T-cell epitope vaccines and immunotherapy [90,93]. Unfortunately, at present, there are no HLA transgenic guinea pig models with which to study the immune response that controls the lifecycle of HSV-2 and test vaccine efficacy [93]. In addition, the necessarily exclusive use of female guinea pigs in this model makes it impossible to account for sex as a biological variable.

### 2.4. Tree Shrews

The squirrel-like tree shrew has been used by a few groups as a model of HSV latency since the 1970s [116]. Tree shrews provide a better model for human HSV-1 infection than the other rodents [117]. This is due to close similarities to humans in their anatomy, neurodevelopment, and immune and metabolic systems [118]. Tree shrews also have the advantage of short life and reproductive cycles, and high reproductivity compared to primates [119]. Unlike mice, in which HSV does not spontaneously reactivate, tree shrews experience reactivation and recurrent herpetic mucosal lesions similar to humans [120]. Tree shrews represent a more humanlike model in which to study HSV infection, perhaps providing valuable insights that could not be gleaned from other animal models. A study of latent HSV-1 in the tree shrew trigeminal ganglion found significant differences in the gene expression of ICP0, ICP4, and LAT compared to mice [121]. Tree shrews had undetectable levels of ICP4, correlating with a weaker acute infection but higher levels of ICP0 and LAT during latency than mice [121]. The same group also found that the route of HSV-1 infection into the CNS was predominately through the olfactory bulb in tree shrews but through the brain stems in mice [122]. Tree shrews do have limitations as models for HSV infection, including the lack of an inbred genetic background, and currently limited research techniques and materials such as gene knockout and tree shrew-specific antibodies. The complete genome of the tree shrew has been published, and remarkably they appear closer related to humans than to rodents. Due to this close relation, some molecular tools, such as anti-human antibodies, cross-react with tree shrew proteins [118]. As this species is increasingly used in cancer research to model human carcinogenesis, more tools are in development, carrying promise for the tree shrew as an HSV infection model [123].

### 2.5. Cotton Rats

The cotton rat, a common New World rodent, is a well-established model of human viral diseases that is distinct from laboratory mice and rats [100]. Unlike murine models, the cotton rat is susceptible to other human viruses, such as respiratory syncytial virus (RSV) and measles, in addition to HSV. Infection in the cotton rat closely mimics human oral HSV-1 infection (herpes labialis) and provides an alternative model for genital HSV-2 infections [100,103]. Notably, the cotton rat does not need hormonal manipulation for vaginal HSV infection that murine models rely upon [100]. Another advantage of the cotton rat is the spread of virus to organs targeted in disseminated human HSV disease, including liver, lungs, and kidneys [100]. Cotton rats are also prone to spontaneous reactivation, similar to a human infection, with self-limited small lesions. The cotton rat model has additional advantages over other spontaneous reactivating models of HSV. These include the increasing availability of immunological reagents, the potential for vertical and horizontal transmission as seen for RSV, and the ability to study coinfections [100,104,105,106].

### 2.6. Hamsters

Although the Syrian golden hamster is not a common animal model to study HSV, it has been used as a human model for viral infections such as alphaviruses, SARS-CoV, flaviviruses, and bunyaviruses [91]. Overall, the low cost, short reproductive cycle, ease of handling, and ability to recapitulate disease progression in humans make the Syrian golden hamster a suitable alternative animal model. Hamsters are outbred, which provides a model with more genetic diversity. Their immune system, however, is significantly different from that of humans [90]. Additionally, there are limited reagents available to study the immune response, as well as insufficient characterization of immune-specific markers in hamsters [90]. Since the immune response cannot be evaluated, this hinders its use as a model for vaccine and therapeutic drug development [91].

### 2.7. Non-Human Primates

While primates are the most expensive animal model with the most ethical concerns, non-human primates (NHPs) have a genetic background, anatomy, physiology, and immune response that is highly similar to that of humans [124]. Among the New World NHPs used to study HSV are the owl monkey and the common marmoset. These NHPs have high susceptibility to HSV and pathology resembling an HSV infection in human newborn infants [125,126,127]. While the owl monkey has been recently harder to obtain, the common marmoset is bred in facilities within the United States and is readily available [128]. The rhesus macaque, an Old World NHP, has also been used as a model for both HSV-1 and HSV-2 infections. HSV-1 infection progresses similarly in humans and macaques, in which the virus is transmitted to nervous tissues by retrograde transport along the peripheral nerves and establishes latency in the trigeminal ganglia [129,130]. This NHP model develops the clinical manifestations and pathological features observed in humans during an HSV-1 infection, such as recurrent vesicular lesions, increased body temperature, and viral shedding in the eyes or mouth [131]. The similar disease progression in NHPs and humans makes NHPs a valuable, though sparingly used, model to study viral pathogenesis and latency.

## 3. Primary Neuron Models of Latency

While in vivo models are critical to decipher the complex relationship between HSV pathogenesis and host immunity, primary sensory or sympathetic neuron cultures have yielded invaluable insights into the mechanistic aspects of HSV latency and reactivation in ex vivo models. In vivo models have limitations such as long turnaround times and drug toxicity effects, and for neuronal studies, they have difficulty selectively targeting CNS and PNS tissues with drugs and vectors. Moreover, the cell composition of the typical ganglion from mice or rabbits is only ~10% neurons, only 10–20% of which are latently infected [132]. A data analysis from such low numbers of latently infected neurons on a background of uninfected neurons is complex. Primary neuronal cultures, on the other hand, have a shorter setup time and facilitate the access of viruses and other experimental reagents [56]. They can be monitored in real time during infection with HSV-1-expressing fluorescent proteins, and they can be readily manipulated by molecular techniques such as gene delivery or RNAi [56,133]. While primary neurons from prenatal and postnatal rats or adult mice are historically the best characterized in vitro/ex vivo models to study HSV latency and reactivation, other species (humans, rabbits, NHPs, and chickens) have also been used [46,134,135,136,137,138]. Since most studies have focused on mice, we confine our descriptions within this review to mouse studies unless otherwise noted. These models fall into two main categories discussed below. In the first, naïve neurons are isolated and then infected in vitro in the temporary presence of replication inhibitors such as acyclovir and interferon to promote viral latency [102]. In the second, HSV infection and ensuing latency are established in the animal prior to ex vivo isolation of a mixed culture of latently infected and uninfected neurons.

### 3.1. Sensory and Sympathetic Neurons

The two main classes of primary neurons used for the in vitro study of HSV latency are sensory and sympathetic neurons, which are isolated from ganglia. Sensory neurons transmit signals in an afferent manner, that is, from the PNS to the CNS. Sensory neurons relay important environmental information from the skin and internal organs to the brain or spinal cord [139]. The sensory tissues naturally and most commonly infected with HSV are the trigeminal ganglia (TG) and dorsal root ganglia (DRG), although the geniculate ganglia and vestibular ganglia can also support HSV infection [140,141]. The TG consist of a bundle of sensory neurons surrounded by satellite glial cells, microglia/macrophage-like cells, and Schwann cells [142]. TG neurons are specialized pseudounipolar neurons located at the base of the skull that project directly to the brain stem or upper regions of the spinal cord [143]. The TG have three nerve branches that travel along the side of the head and are responsible for detecting and responding to pain, touch, and temperature stimuli from the face [144]. Like the TG, the DRG are a collection of pseudounipolar sensory neurons surrounded by satellite glial cells that carry mechanical, chemical, and thermal signals from the PNS to the CNS [145]. DRG neurons arise from the dorsal root of the spinal nerves to innervate the body [145]. Sensory neurons are further classified based on their neurotrophic dependency, myelination, neurofilament type, and neuropeptide expression [146,147]. The establishment of HSV-1 latency differs in two subtypes of sensory neurons, classified either as neurofilament heavy-positive (NefH^+^) or -negative (NefH^−^). NefH^+^ neurons, specifically those co-expressing the calcitonin gene-related peptide α (CGRP^+^), have higher LAT promoter activity and are infected at a decreased rate compared to NefH^-^ neurons [148,149,150,151]. This could be due to reduced axonal retrograde transport of HSV-1 to the cell body of NefH^+^ neurons, thus benefitting the host anti-viral response and promoting expression of LAT through miRNAs, chromatin modification, and anti-viral activity [148].

While sensory neurons are the most common site of HSV latency in humans, latent HSV infections are also established in sympathetic neurons [152,153,154]. In contrast to sensory neurons, sympathetic neurons are motor neurons that relay signals in an efferent manner from the CNS to the PNS, muscles, or glands [155]. A natural center of HSV infection and the most common sympathetic neuron model to study HSV is the superior cervical ganglia (SCG). The SCG consists of a group of sympathetic neurons that are integral to the autonomic nervous system as the primary source of sympathetic innervation of the face and head. Since the location and size of rodent SCG facilitate rapid isolation and dissociation, SCGs are a common source of neurons for in vitro HSV studies [156]. The isolation of SCG neurons produces relatively homogenous cultures of neurons that are capable of establishing and recapitulating HSV latency and reactivation in response to stress, similar to that in animal models [56,133].

HSV reactivation often follows physical or psychological stress, through mechanisms involving stress hormones (such as epinephrine and cortisol), NGF-deprivation, forskolin, and intracellular cAMP [56,136,157,158,159,160]. Interestingly, epinephrine, which is regulated by the sympathetic nervous system, can induce the reactivation of HSV-1 both in vivo and in vitro, yet it does not induce the reactivation of HSV-2 [161,162]. Furthermore, epinephrine selectively stimulates the reactivation of HSV-1 in murine sympathetic, but not sensory, neurons in vitro [135]. Cortisol, which suppresses the immune system and regulates metabolism, can induce reactivation of both HSV-1 and HSV-2 in vivo and in vitro models [135,159,163]. Similar to epinephrine, cortisol induces reactivation selectively in sympathetic neurons [135]. The differing effects of HSV type and neuron source on reactivation could result from virus type-specific factors and/or varying expression of epinephrine and cortisol receptors on sensory and sympathetic neurons [135,163,164,165]. Further research, using both in vivo and in vitro models, will help to determine what factors influence preferential latency reactivation in sensory and sympathetic neurons.

### 3.2. Ex Vivo Explant Models of Latency

Latently infected ganglia explants, most commonly from mice (but also from rabbits, guinea pigs, tree shrews, humans, and NHPs), have been used as an ex vivo model to study the molecular events occurring upon HSV reactivation [122,166,167,168,169,170,171]. In contrast to in vitro models, ex vivo neuronal cultures harbor latent HSV infections established naturally (i.e., without DNA replication inhibitors). Rather than relying on drug treatments, reactivation typically results from the physical disruption of axons during the excision process. This neuronal axotomy leads to an influx of intracellular calcium, resulting in increased intracellular cAMP, which interacts with the LAT promoter region to induce reactivation [172,173]. In addition to excision, reactivation can also be induced in vivo (prior to dissection) through hyperthermia, skin abrasions, or CD8^+^ T-cell depletion [174,175,176]. While commonly focused on the PNS, ex vivo latency experiments using brain stems and olfactory bulbs from tree shrews and mice have demonstrated a difference in HSV reactivation between the CNS and PNS [122,177]. In comparison to latent TG infections, latent infections in brain stems and olfactory bulbs reactivate at a lower frequency [122]. Studies conducted with latently infected ganglia explants have led to the discovery of the mechanisms and roles of a broad array of HSV genes in regulating HSV latency and reactivation. For example, through the use of LAT-deficient HSV-infected explants, it was shown that LAT is associated with latency establishment, maintenance, and reactivation [60,178,179]. ICP0-null and TK-null latently infected explants, on the other hand, demonstrated that ICP0 and TK are not required for latency establishment but are necessary for efficient reactivation [60,180]. Additional studies have also demonstrated that the reactivation of latently infected ganglia explants results in decreased LAT expression and decreased LAT enhancer histone acetylation prior to increasing ICP0 promoter acetylation and transcription [181], supporting the hypotheses that LAT functions in an antisense mechanism toward IE promoters and that chromatin remodeling can regulate latency and reactivation. Ganglia explants have also been used to investigate the kinetics of HSV gene expression during reactivation [182]. In contrast to lytic infections, reactivation in explant ganglia results in disordered, rather than sequential, expression of viral genes [182,183]. This could suggest that the establishment of, or reactivation from, latency is not dependent on the specific expression of the IE genes [183].

Latently infected ganglion explants, in contrast to in vitro primary neurons, also provide a model to determine how various immune mechanisms influence HSV latency establishment and reactivation. Experiments using mouse TG explants have demonstrated that CD8α^+^ dendritic cells rather than CD8^+^ T-cells contribute to enhanced HSV-1 latency and recurrences [184]. In addition, studies using latently infected sensory and sympathetic ganglia have demonstrated how different signaling pathways modulate HSV latency. For instance, TG explant studies have determined that activation of the glucocorticoid receptor through the synthetic corticosteroid dexamethasone induces HSV reactivation [185,186]. Alternatively, NGF is important for maintaining latency, and the deprivation of NGF from latently infected explant cultures results in accelerated reactivation [183].

Latently infected ex vivo explants have been instrumental in advancing our understanding of the key viral genes and molecular mechanisms involved in HSV latency and reactivation. Although ex vivo explants have factors that more closely mimic the natural processes in the HSV lifecycle during a human infection, such as establishing latency without DNA replication inhibitors and reactivating from stress stimuli, there are limitations to this model. One such disadvantage of this model is that latency is only established in 10–20% of the neurons in the ganglia, and reactivation is induced in 5–10% of those latently infected neurons [187]. Since stresses in addition to excision can have an additive impact on reactivation, modifications to ex vivo protocols, such as antibody-mediated depletion of NGF, can be used to increase the incidence of HSV reactivation in these neurons [188].

## 4. Differentiated Cell Lines

Primary neuron models consist of a heterogeneous population of neurons co-isolated with supporting cells, including glia cells, as well as immune cells such as microglia and resident T-cells [102]. HSV latency and reactivation differ in such heterogenous populations depending on the unique cellular constituency and reactivation stimulus applied [189]. Culture heterogeneity complicates analyses of host and viral responses since subsets of cells vary in their susceptibility to infection and ability to support latency establishment [102]. An ideal model to study the establishment and maintenance of HSV latency would consist of a homogenous monoculture of neurons. A homogenous neuron model can be obtained through cell lines that can be differentiated into mature, functional, and specific subtypes of neuron-like cells. In comparison to primary neurons, neuronal cell lines are more cost effective, are easier to transfect, can be directly gene edited, and allow for high-throughput studies due to their proliferative nature, unlike primary neurons, which are terminally differentiated. Another distinct advantage of using cell lines is the avoidance of ethical and regulatory concerns surrounding the use and treatment of animals. That said, certain embryonically derived human cell lines may also raise ethical and regulatory issues. The three main categories of cell lines used as HSV latency models are neural crest-derived, neuroblastoma-derived, and induced pluripotent stem cells (iPSCs). Each of these models has advantages and disadvantages, as summarized in Table 2, and careful consideration should be given when choosing one as a model for HSV latency.

### 4.1. Rodent Cell Lines

Derived from a single-cell clonal line from a transplantable rat adrenal pheochromocytoma, the PC12 cell line is neural crest-derived and reversibly differentiated into neurons through the addition of NGF [190]. Differentiation begins within a week of NGF exposure and progresses over several weeks, during which the cells cease division and produce axonal processes similar to sympathetic neurons [190]. If NGF is removed during this period, axonal processes are degraded, and cell division resumes within 72 h [190]. While PC12 cells synthesize and store dopamine and norepinephrine similar to rat adrenal neurons, epinephrine is not synthesized or induced in response to treatment such as dexamethasone [190]. Although PC12 cells are similar to mature sympathetic adrenal neurons, they lack expression of the functional glutamate receptor *N*-methyl-d-aspartate (NMDA) [191]. PC-12 cells are permissive to HSV-1 infection and support the establishment of latent infection and viral reactivation, both induced and spontaneous [192]. While the use of DNA replication inhibitors is required to establish viral latency in this cell line, the inhibitors are not required to maintain latency [192].

The neuroblastoma cell line Neuro-2A is another common neuron model, derived from the mouse neural crest [194]. Neuro-2A cells quickly differentiate into neurons within days after the activation of extracellular signal-regulated kinase (ERK) through addition of forskolin, retinoic acid, 2,4-dinitrophenol, or serum deprivation [195,196,197]. While Neuro-2A cells become dopaminergic in the presence of dibutyryl cAMP (dbcAMP), or cholinergic in response to retinoic acid, they are more similar to adrenal gland cells and sympathetic neurons like PC12 cells [194,198]. A cell line similar to Neuro-2A, C1300, derives from a different part of the same mouse neuroblastoma as Neuro-2A [199]. Both Neuro-2A and C1300 cells have been utilized as models of different aspects of the HSV lifecycle, including acute infection and latency. One caveat of using immortalized cell lines is that they differ considerably from primary neurons, due in part to genetic drift and mutations accumulating over recurrent passaging [191,200]. One example is the dampened sensitivity of Neuro-2A cells to neurotoxins, thought to result from an altered density of cell membrane receptors and ion channels, similar to a lack of NMDA receptors in PC12 cells [200,226]. Notwithstanding these caveats, these cell types have allowed useful contributions to our understanding of HSV latency [64,193,199].

### 4.2. Human Cell Lines

Since HSV is a human virus, human neurons are the ideal model to study neurotropic infection. While primary human neurons can be obtained from post-mortem tissue, this is not a generally feasible source of neurons with which to model viral infection [227]. Not only is viable post-mortem nervous tissue difficult to obtain, but also such neurons are very commonly latently infected with neurotropic herpesviruses, including VZV and HSV, thus precluding the study of HSV in a controlled manner [228]. Most commonly, human neurons for HSV studies are derived from the differentiation of embryonic stem cells, induced pluripotent stem cells, neuronal stem cells, and immortalized neuron cell lines. These differentiated or immortalized neurons can be used to study the mechanisms that influence HSV latency and reactivation in the physiologically relevant setting of the human neuron.

Lund human mesencephalic (LUHMES) cells are human embryonic neuronal precursor cells, which express a tetracycline-regulatable (Tet-off) v-myc transgene that, when activated, causes differentiation into mature dopaminergic neurons [132,203]. These cells are derived from a subclone of the MESC2.10 cell line, which originated from 8-week-old human ventral mesencephalic tissue and was characterized at the Lund University in Sweden [204,229]. Since LUHMES cells function under a Tet-off system, they remain proliferative and can be propagated routinely prior to differentiation by tetracycline. LUHMES cells thus are useful for large-scale and high-throughput studies, pointing to a common limitation of primary neuron models. Another significant advantage of LUHMES cells is that differentiation into mature neurons takes place within 5 days after tetracycline addition, compared to the weeks or months of maintenance for in vivo, ex vivo, and primary neuron cultures [132]. These cells support HSV-1 infection, and latency is established and maintained after two days of ACV treatment, as determined by decreased lytic gene expression and increased LAT expression six days post-infection [132]. This provides a distinct advantage compared to other in vitro and primary neuron cultures that require five days of ACV treatment in order to establish and maintain latency. Latency in LUHMES cells is similar to that in latently infected ganglia, in which most cells harbor the HSV-1 genome yet only a fraction express detectable LAT [132,230,231]. While spontaneous reactivation is uncommon, viral reactivation can be induced upon the inhibition of phosphoinositide 3-kinase (PI3K) [132].

Derived from the DRG of a first-trimester fetus, the HD10.6 immortalized cell line also uses a doxycycline inducible Tet-off oncogene to drive differentiation into neonatal sensory-like neurons with nociceptive properties [201,202]. While the establishment of replicative HSV infection in HD10.6 neurons is delayed relative to primary neurons, they do support latent infection in the presence of ACV at a low multiplicity of infection (MOI) [202]. Although reactivation can be induced through combined superinfection with UV-inactivated virus and NGF deprivation, stressors commonly used to induce reactivation in other neuronal models are ineffective in HD10.6 cells [202]. The unusual difficulty in eliciting reactivation could be related to various factors, including lower levels of LAT, or mutations accumulated during immortalization or cell passaging that causes attenuation of neurotrophic signaling [102].

The cell line SH-SY5Y is an embryonic stem cell line derived through consecutive sub-clonings of the SK-N-SH neuroblastoma cell line commonly used in neurological studies [205]. Undifferentiated SH-SY5Y cells are non-polar and resemble neuroblast-like cells and immature catecholaminergic neurons [206,207]. A unique advantage of SH-SY5Y cells is that they can differentiate into homogenous populations of cholinergic, adrenergic, or dopaminergic neurons depending on their exposure to neurotrophins [207]. They are frequently differentiated into cholinergic neurons through exposure to retinoic acid, but they can develop a dopaminergic phenotype when retinoic acid is used in combination with phorbol esters [208]. When cultured with phorbol esters alone, such as 12-*O*-tetradecanoyl-phorbol-13 acetate (TPA), they will differentiate into adrenergic neurons [209]. In the context of HSV infection, differentiated SH-SY5Y cells are highly permissive for the replication of HSV-1. In these cells, viral replication still occurred in the presence of ACV at an MOI 1000-fold lower than what is sufficient for controlling infection in rat SCG models [46,102]. This is perhaps due to a more permissive environment in human neurons, the neuron subtype, or the source of neurotropic support [46]. Although the virus is not controlled at high MOIs, after six days in the presence of ACV and interferon-α at a low MOI, the virus is able to establish a stable latency-like infection [102]. This latent infection, characterized by increased LAT and decreased lytic gene expression, can be maintained for several weeks in the absence of ACV before induced reactivation with a stimulus such as sodium butyrate. While SH-SY5Y cells can be used as a human neuronal model to study HSV-1, the process of differentiating SH-SY5Y requires the stepwise addition of inhibitors and growth factors over a month in order to mimic neurogenesis. This can result in high variability of neuronal subtypes between labs using the same cell line and protocols [102,210,232,233,234]. Due to these limitations, it is becoming more common to use neuronal transcription factor-driven differentiation to induce rapid and reproducible neurogenesis.

Directed neuronal differentiation, through constitutive or induced overexpression of transcription factors, bypasses the progenitor stage in differentiation, resulting in significantly faster neuron generation [211,212,213]. In vivo neuron generation is a highly complex process driven by both intrinsic and extrinsic factors, which ultimately influence the neuronal subtype. The determination of which transcription and growth factors are essential to neurogenesis has made it possible to differentiate iPSCs into specific subtypes [210]. The transcription factors from the neurogenin family (NGN), consisting of NGN1, NGN2, and NGN3, are of particular interest. This family of transcription factors is expressed throughout the nervous system and affects the commitment of progenitors to neurons by promoting subtype-specific gene expression [210]. In particular, NGN2 is a key regulator of differentiation to glutamatergic, dopaminergic, motor, and sensory neurons, but it inhibits differentiation into GABAergic neurons, oligodendrocytes, and astrocytes [210]. This master regulator role for NGN2 in neurogenesis has made it a common focus for overexpression to rapidly yield a variety of mature neurons in a reproducible manner [211,212,213,214,215,216]. The overexpression of NGN2 and related neuronal transcription factors provides a scalable, high-throughput, rapid, and reproducible human neuronal model for HSV studies. While NGN2 has been widely used to generate differentiated neurons, other transcription factors can efficiently generate a homogenous neuronal population [217]. In particular, NGN3 overexpression can lead to rapid differentiation of iPSCs into mature sensory neurons [64,217]. These differentiated human neurons are permissive to HSV-1, and under the influence of ACV, HSV can establish a quiescent latency-like state that can be stimulated with histone deacetylase inhibitors to induce HSV reactivation.

### 4.3. Organoid Cultures

A variety of models have been used to gain insight on HSV latency and reactivation, each of which has advantages and disadvantages that should be carefully considered when planning experiments. While some in vitro cell lines have the advantage of a human-like model, they lack the complex multicellular and immune interactions found in vivo. For instance, ganglia consist primarily of non-neuronal cells, such as satellite and immune cells, that could influence mechanisms within neurons to control viral gene expression, the establishment and maintenance of latency, and virion production [187]. Neurons produce low levels of IFN, relying on support cells to produce IFN or inflammatory cytokines in response to an HSV-1 infection [235,236]. In order to accurately recapitulate HSV-1 infections, a model that captures both multicellular and immune interactions in the context of a human cell source is especially needed. One way to achieve this is through three-dimensional (3D) organoids, in vitro cell aggregates derived from stem cells which exhibit self-organization and organ-like functionality [218,219]. Three-dimensional organoids can mimic aspects of the composition and architecture of the brain, with intercellular and cell-to-extracellular matrix (ECM) interactions that are lacking in 2D neuron cultures [220]. The differentiation protocols used to generate brain organoids are relatively cost-effective, are scaffold-free, and produce organoids with a consistent diameter. Three-dimensional organoids may thus provide a promising, highly malleable, complex cellular model to study host–pathogen interactions that does not suffer the limitations of in vivo models.

Human iPSC-derived brain organoids are permissive to HSV-1 and express LAT in the outer layers without the need for antivirals that are necessary to drive latency in 2D neuron cultures [220]. One caveat of this model is a low rate of reactivation in latently infected organoids, which differs from a high reactivation efficiency characterized by iPSC-derived neuron cultures. When infected in the presence of antivirals, however, HSV-1 spontaneously reactivates in 17% of brain organoids after the removal of antivirals [220]. This discrepancy could be due to the different cell-to-cell and cell-to-ECM interactions and the gene expression variations between 3D and 2D environments [221,222]. This emphasizes the importance of a model that most accurately mimics the human nervous system for the study of HSV-1 latency and reactivation. While brain organoids provide a model that mimics the architecture and multicellular interactions of the human brain, they cannot account for systemic aspects and the role of the adaptive immune response in HSV-1 infections. To address this, a brain organoid-on-a-chip model can be used to allow for precise control over the neuron microenvironment through microfluidics that mimics the blood–brain barrier, fluid flow in cerebrospinal spaces, and vascular structures [223,224]. This is achieved through the gravity-driven, continuous flow of nutrients and the removal of metabolic waste to and from the organoids. Additionally, using a 3D-printed microfluidic device, neurovascular brain organoids capable of permeability and perfusion can be used to deliver membrane-diffusible molecules from blood vessels to the organoids [223]. Such fluid-flow models in combination with 3D organoid cultures may better mimic the human neuro-microenvironment, particularly in light of different stimuli, including those involved in adaptive immunity [225].

## 5. Concluding Remarks

Our understanding of HSV latency has been obtained through clinical observations in humans and a variety of in vivo, ex vivo, and in vitro model systems. One such important clinical observation is that individuals with HSV-1 genital infections, in comparison to HSV-2, experience less frequent reactivation and asymptomatic shedding [6]. This could simply result from differences between HSV strains and neuronal subtypes which can affect disease progression, as discussed previously. Additionally, immune responses likely differ between ganglion type and anatomy, with distinct local immune responses. This difference in immune response may potentially be influenced by sex hormones such as estrogen and progesterone [237]. Several studies have shown that estrogen/progesterone can alter T-cell and cytokine function and increase susceptibility to HSV infection and reactivation from latency [101,238,239]. Further research is necessary to determine how the immune responses are different in TGs and DRGs, and between HSV-1 and HSV-2, and whether those differences affect mechanisms involved in HSV latency and reactivation.

The in vivo models have been instrumental in determining viral–host interplay that result in latency, while the in vitro models have been more valuable in elucidating molecular pathways involved in the establishment, maintenance, and reactivation of latency [87]. Unfortunately, each of the current models has limitations, such as immune responses that differ significantly from humans, ethical concerns, cost, time, and reagent availability. Additionally, careful consideration must be taken to ensure reproducibility and limited variability, using different in vivo and in vitro models. The variability seen within the field could be a result of the different species, cell types, neuronal subtypes, and HSV strains used. Both in vivo and in vitro studies have shown that there are strain-specific differences in the virulence and latency/reactivation of HSV-1 [240]. There is also evidence that cell type-specific factors, in addition to virus-specific factors, contribute to HSV-1 pathogenesis [241]. Although there has been concern about the divergence of common HSV laboratory strains over time, this can be mitigated through the low passage of laboratory strains and correlative use of clinical isolates [242,243]. Learning about strain-specific differences in latency and reactivation could yield important information regarding the roles of specific viral genes in pathogenesis. Therefore, to ensure standardization within the field, multiple in vivo and in vitro model systems and HSV strains should be used in combination to validate both the current and past findings.

While new animal models and technological advancements have been made to improve current HSV models, there still remains the need for a model that more accurately replicates human HSV infections in a time- and cost-efficient manner. This would require a model utilizing human cells that can address the immune and multicellular interactions during an HSV infection. The field is currently shifting to incorporate animal systems, as well as human cell lines or 3D organoid models to study latency mechanisms and host–virus interactions [56]. These new technologies and model systems should provide further insight regarding some ongoing questions in the field: Why is HSV latency selectively established in neurons? What host factors and pathways influence latency and reactivation? What host and viral mechanisms control gene expression during latency? Can we use these models to identify points in the latency/reactivation pathway that would represent therapeutic targets to alter the establishment, maintenance, or reactivation of latency? Given the broad array of outcomes following HSV infection in humans, it seems unlikely that one model could mimic all aspects of HSV pathogenesis and address these questions. By utilizing salient aspects of different models, we can integrate information to collectively and reliably predict the determinants of HSV pathogenesis in humans.

## Figures and Tables

**Table 1:** In vivo latency model comparison.

Model	Hamster 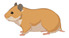	Mouse 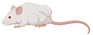	Rat 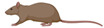	Guinea Pig 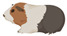	Rabbit 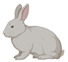	Tree Shrew 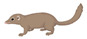	Non-Human Primate 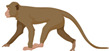
**Reagent** **availability**	Low	High	Medium	Low	Medium	Low	High
Overall cost *	$	$	$	$	$$	$$$	$$$$$
**Similarity to human immune response**	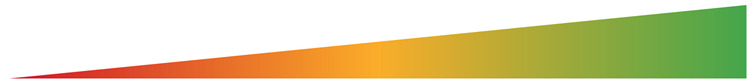						
**Spontaneous** **reactivation**	Unknown	No	Yes	Yes	Yes	Yes	Yes
**References**	[90,91]	[92,93,94,95,96,97,98,99,100,101,102]	[100,103,104,105,106]	[90,93,107,108,109]	[93,110,111,112,113,114,115]	[116,117,118,119,120,121,122,123]	[124,125,126,127,128,129,130,131]

* Determined based on estimated market rate and per diem in the United States per animal: $ (under USD 100), $$ (under USD 300), $$$ (under USD 500), and $$$$$ (over USD 5000). Graphics created with BioRender.com.

**Table 2:** In vitro latency model comparison.

	Primary Neurons	Ex Vivo Explant	PC12	Neuro-2A/C1300	HD10.6	LUHMES	SH-SY5Y	iPSC	Brain Organoid
Model origin *	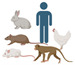	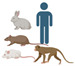	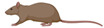	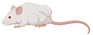	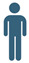	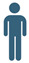	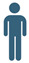	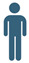	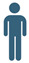
**Throughput**	Low	Low	High	High	High	High	High	High	High
**Time**	Months	Months	Months	Days	Days	Days	Months	Days	Months
**Neuron subtype**	Sympathetic or Sensory	Sympathetic or Sensory	Sympathetic	Sympathetic	Sensory	Dopaminergic	Cholinergic, Adrenergic, or Dopaminergic	Glutamatergic, Dopaminergic, Motor, or Sensory	Central Nervous System
**Reactivation**	Yes	Yes	Yes	Yes	Yes	Yes	Yes	Yes	Yes
**References**	[46,56,133,134,135,136,137,138,139,140,141,142,143,146,147,148,149,150,151,152,153,154,155,156,157,158,159,160,161,162,163,164,165]	[60,122,166,167,168,169,170,171,173,174,175,176,177,178,179,180,181,182,183,184,185,186,187,188]	[190,191,192,193]	[64,194,195,196,197,198,199,200]	[102,201,202]	[132,203,204]	[46,102,205,206,207,208,209]	[64,210,211,212,213,214,215,216,217]	[218,219,220,221,222,223,224,225]

* Graphics created with BioRender.com.

## Data Availability

Not applicable.
